# PKR inhibitors suppress endoplasmic reticulum stress and subdue glucolipotoxicity-mediated impairment of insulin secretion in pancreatic beta cells

**DOI:** 10.3906/biy-1909-20

**Published:** 2020-04-02

**Authors:** Abdullah YALÇIN, Gülçin ŞARKICI, Umut Kerem KOLAÇ

**Affiliations:** 1 Department of Medical Biology, Faculty of Medicine, Adnan Menderes University AYDIN TURKEY

**Keywords:** Type 2 diabetes, protein kinase R, ER stress, β cell degeneration

## Abstract

Type 2 diabetes mellitus is characterized by insulin resistance and hypersecretion of insulin from the pancreas to compensate for decreased insulin sensitivity in the peripheral tissues. In later stages of the disease insulin-secreting beta cell degeneration commences and patients require insulin replacement therapy in order to accomplish proper regulation of their blood glucose. Endoplasmic reticulum (ER) stress in the beta cells is one of the factors contributing to this detrimental effect. Protein kinase R (PKR) is a cellular stress kinase activated by ER stress and contributing to degeneration of pancreatic islets. In order to determine whether inhibition of PKR activation by specific small molecule inhibitors of PKR ameliorates pancreatic insulin secretion capacity, we treated beta cells with two imidazole/oxindole-derived inhibitors of PKR kinase, imoxin (C16) and 2-aminopurine (2-AP), in the presence of ER stress. Our results demonstrate that PKR inhibition suppresses tunicamycin-mediated ER stress without altering the insulin production capacity of the cells. Palmitic acid-mediated suppression of insulin secretion, however, was subdued significantly by PKR inhibitor treatment through an ER stress-related mechanism. We suggest that PKR inhibitor treatment may be used to increase the insulin secretion capacity of the pancreas in later stages of diabetes.

## 1. Introduction

Type 2 diabetes (T2D) is a chronic disease that affects millions of people around the world negatively and causes various micro- and macrovascular complications in the body (Drong et al., 2012). In recent years, the prevalence of T2D in developed and developing countries has increased drastically, making the disease one of the most important threats to public health (Whiting et al., 2011). Type 2 diabetes is usually characterized by insulin resistance that develops after 30 years of age, compared to type 1 diabetes (T1D), which occurs as an autoimmune disease and causes insulin deficiency generally during early childhood (Cnop et al., 2005). Insulin-sensitizing drugs that relieve insulin resistance and reduce its detrimental effects are frequently used in early stages of the disease (Peters, 2013). Generally, these conventional drugs exhibit well-documented blood glucose-lowering actions and are preferred for their effectiveness in controlling micro- and macrovascular complications of diabetes. Given the progressive nature of the disease, conventional insulin-sensitizing drugs remain effective in controlling blood glucose levels for a while. In the later stages of the disease, however, gradual cellular degeneration of insulin-producing cells of the pancreas commences and the effectiveness of insulin-sensitizing drugs decreases significantly (Tibaldi, 2007; Tibaldi and Rakel, 2007). Typically, during these aforementioned stages insulin secretagogues, the drugs that stimulate insulin secretion from the pancreatic cells, are utilized combined with glucose-lowering pharmaceutics. However, this combination therapy has little to no effect in preventing the eventual failure of pancreatic insulin production. In ultimate stages of the disease, pancreatic insulin production and secretion becomes so insufficient that clinicians begin so-called insulin replacement therapy, supporting patients with exogenous insulin hormone analogues (Peters, 2013). Due to its relative difficulty of application and the adverse effects on the life quality of the patients, insulin therapy is used only after pancreatic insulin production and secretion fail to achieve effective control of blood glucose levels (Raskin et al., 2005). 

The endoplasmic reticulum (ER) is a crucial organelle that controls synthesis and folding of secreted proteins, synthesis of lipids, and maintenance of proper intracellular Ca2+ balance (Hotamisligil 2009). Some critical observations in the last decade put the ER in the spotlight of clinical research. These findings suggest a close association between metabolic diseases and increased ER stress in the metabolically important tissues (Özcan et al., 2004; Hotamisligil, 2010). ER stress is usually triggered by accumulation of unfolded protein load in the ER lumen, which compromises the synthesis and secretion activity of the organelle (Hotamisligil, 2010). In this scenario, the cell initiates a relief mechanism called unfolded protein response (UPR), leading to the halting of global protein synthesis, increasing the chaperone machinery and thus the folding capacity of the ER, and lastly clearance of the unfolded proteins from the ER lumen (Hotamisligil, 2009, 2010). UPR achieves these through at least three sensory/signal transduction pathways, namely phosphorylation of eukaryotic initiation factor 2 alpha subunit (eIF2α) by ER membrane kinase PKR-like endoplasmic reticulum kinase (PERK), cellular modification of x-box binding protein-1 (XBP-1) mRNA through inositol-requiring enzyme 1 (IRE1) protein, and nuclear localization of ER membrane bound transcription factor activating transcription factor 6 (ATF6) (Marciniak and Ron, 2006). Nevertheless, in some cases the ER protein load cannot be cleared efficiently by UPR pathways or ER stress persists in a chronic fashion, as in obesity and related metabolic disorders. In such instances, UPR, through activation of the C/EBP homologous protein (CHOP) transcription factor, leads to programmed cell death and disposal of the troubled cells (Lakshmanan et al., 2013). Recent studies indicated that this chronic ER stress may be involved in the β cell degeneration during the late phases of diabetes and the gradual decrease of insulin secretion capacity of the pancreas (Engin et al., 2014). Pancreatic beta cell survival and insulin secretion activity are highly susceptible to glucolipotoxicity. ER stress can be consistently induced by free fatty acid palmitate (PA) in the presence of excessive concentrations of glucose, which in turn induces beta cell apoptosis and islet degeneration (Barlow et al., 2016; Liu et al., 2019).

Protein kinase R (PKR) is one of the cytoplasmic regulators of protein synthesis machinery. Like PERK, PKR catalyzes phosphorylation of eIF2α in response to various cellular stress signals. The canonical activator of PKR is double-stranded RNA, as a defense mechanism against pathogens such as RNA viruses (Rothenburg et al., 2009; Carpentier et al., 2016). However, PKR is also activated by other factors such as heat shock proteins, growth factors (platelet-derived growth factor, etc.), heparin and cytokines, reactive oxygen species, and ER stress-inducers like tunicamycin and thapsigargin (Gil and Esteban, 2000; Garcia et al., 2007; Watanabe et al., 2018). The triggering of ER stress by tunicamycin, which inhibits N-linked glycosylation in the ER, is well documented in insulin-secreting cells (Srinivasan et al., 2005). In this context, the main function of PKR is to contribute to the blocking of protein translation and hence relieve the unfolded protein load of ER by phosphorylation of the eukaryotic initiation factor eIF2α subunit. Nevertheless, substantial increase of PKR phosphorylation activity is also involved in insulin resistance and contributes to the progress of T2D (Nakamura et al., 2010, 2014). Recent studies have shown that activation of PKR disrupts insulin signaling (Udumula et al., 2017) and stimulates insulin resistance in peripheral tissues (Nakamura et al., 2014). 

The aim of this study is to determine the effect of ER stress through PKR activation on insulin secretion machinery using insulin-producing pancreatic beta cells (Beta-TC-6) as a model system. Glucose-stimulated insulin secretion is reported to be impaired by saturated fatty acids (e.g., PA) by a mechanism involving ER stress activation in pancreatic islet cells (Kharroubi et al., 2004). The effect of ER stress on the survival of beta cells is determined by two different approaches to induce ER stress, namely by tunicamycin induction and palmitic acid. Finally, the role of PKR activation in insulin production and secretion is determined by using specific inhibitors of PKR kinase activity, the imidazole-oxindole-derived PKR inhibitor C16 (imoxin) and 2-aminopurine (2-AP) (Ingrand et al., 2007) on beta cells undergoing ER stress.

## 2. Materials and methods

### 2.1. Cell culture and experimental protocol

Mouse Beta-TC-6 (CRL-11506) cells were obtained from the ATCC and grown in DMEM (GIBCO, Sigma-Aldrich Cat. No: D-5546) medium supplemented with 10% fetal bovine serum (GIBCO Cat. No: CP-16-1265), 1% penicillin/streptomycin (Sigma-Aldrich Cat. No: P-4333), and 1% L-glutamine (Sigma-Aldrich Cat. No: G-7513). Cells were incubated in a cell CO2 incubator (NUAIRE) at 37 °C. Stock solutions of PKR inhibitors imoxin (C16) (1 mM, Sigma-Aldrich Cat. No: I9785), 2-aminopurine (250 mM, Sigma-Aldrich Cat. No: A3509), and ER stress-inducer tunicamycin (1 mg/mL, Sigma-Aldrich Cat. No: T7765) were prepared in DMSO (Millipore). Palmitate (sodium salt, Sigma Cat. No: P9767) was solubilized in 90% ethanol (40 mM), heated to 60 °C, and used in a 1:100 dilution in culture medium as previously described (Guo et al., 2007). Cells were first treated with PKR inhibitors, namely imoxin (1 μM) and 2-AP (10 mM) (Nakamura et al., 2014), for 24 h in normal glucose medium (5.5 mM D-glucose). Then tunicamycin was applied for 16 h and cells were incubated in glucose-free medium for 2–3 h. Finally, the cells were taken from glucose-free medium and kept in high-glucose medium (33 mM D-glucose) for 8 h and prepared for protein and RNA isolation. 

In order to induce ER stress by a more physiologically relevant regime, we treated the mouse pancreatic cell line Beta-TC-6 with two different concentrations of palmitate (200 and 400 μM) for 24 h in the presence of 33 mM glucose. We also conducted glucolipotoxicity experiments using immortalized mouse embryonic fibroblast (MEF) cells as a control. Percentages of dead cells in MEF and Beta-TC-6 after PA treatment were determined by Countess Automated Cell Counter (Cat. No: C10227) according to the instructions provided by the manufacturer.

### 2.2. Protein isolation and western blotting

Following incubation, the cells were lysed with RIPA buffer (VRM Life Science Cat. No: N653) containing 1% phosphatase (MedChem Express Cat. No: HY-K0022) and 1% protease inhibitors (MedChem Express Cat. No: HY-K0010). Cells in RIPA buffer were freeze-thawed at 20-min intervals three times and centrifuged at 14,000 × g. The total protein amount of the cells was determined by Bradford assay (Abcam Cat. No: ab102532). SDS-PAGE was performed using proteins loaded equally from each group as a control. After electrophoresis, protein samples were transferred to a PVDF membrane by semidry method. Following transfer, the PVDF membrane was blocked in 1% (w/v) bovine serum albumin for 1 h. The blocked membranes were allowed to incubate with primary antibodies (phospho-elF2α, Elabscience Cat. No: ENP0093, 1:2000) overnight at 4 °C. The membranes were then washed and treated with HRP-conjugated secondary antibodies (antirabbit IgG-HRP, Elabscience Cat. No: SAEP003, 1:5000) at room temperature for 2 h. PVDF membrane was imaged on the UV-Chemi Dot-2 instrument using ECL solution and anti-β-tubulin antibody (Elabscience Cat. No: ENT5051, 1:5000) was used as a loading control. The obtained band images were analyzed with the software program ImageJ.

### 2.3. RNA isolation and RT-PCR

Total RNA isolation from cells was performed using RiboEx Total RNA Isolation Solution” (GeneAll Cat. No: 301-902) and quantified with NanoDrop 8000 (Thermo Fisher). Complementary DNA was then synthesized using a reverse transcription PCR kit (WizScript Cat No: W2211). mRNA was revealed by RT-PCR analysis with RealAmp SYBR qPCR Master Mix and the following XBP-1 mRNA primers: spliced XBP-1 forward: CTGAGTCCGCAGCAGGTG, spliced XBP-1 reverse: TCCTTCTGGGTAGACCTCTGG, unspliced XBP-1 forward: CAGACTACGTGCACCTCTGC, unspliced XBP-1 reverse: GGGTCCTTCTGGGTAGACCT, β-actin forward: AACTGGGACGACATGGAGAA, reverse: GAAGGTCTCAAACATGATCTGG. The relative changes in mRNA expression were achieved by 2–∆∆CT method on a Thermo Fisher Scientific StepOnePlus Real-Time PCR instrument. 

### 2.4. ELISA 

Insulin levels of cells exposed to different doses of tunicamycin (8 μg/mL) were determined using the Elabscience Mouse INS ELISA Kit (Cat. No: E-EL-M2614) and mouse ultrasensitive ELISA assay (ALPCO Cat#: 08-INSMSU-E01) according to the manufacturer’s instructions. Calculated insulin levels were expressed as ng/mL for the Elabscience Mouse INS ELISA Kit or pg/mL for the mouse ultrasensitive ELISA assay (ALPCO Cat#: 08-INSMSU-E01). 

### 2.5. Statistical analysis

All statistical analysis was performed using Prism 5 for MacOS X software (GraphPad Inc.). Averages from multiple (at least 3) independent experiments were compared by two-tailed, unpaired, Student t-tests. P-values lower than 0.05 were considered statistically significant. GSIS insulin secretion curves were compared by two-way ANOVA analysis and P-values lower than 0.05 were considered as significant.

## 3. Results

### 3.1. Endoplasmic reticulum stress is suppressed by PKR inhibitors in pancreatic beta cell line

**Figure 1:**
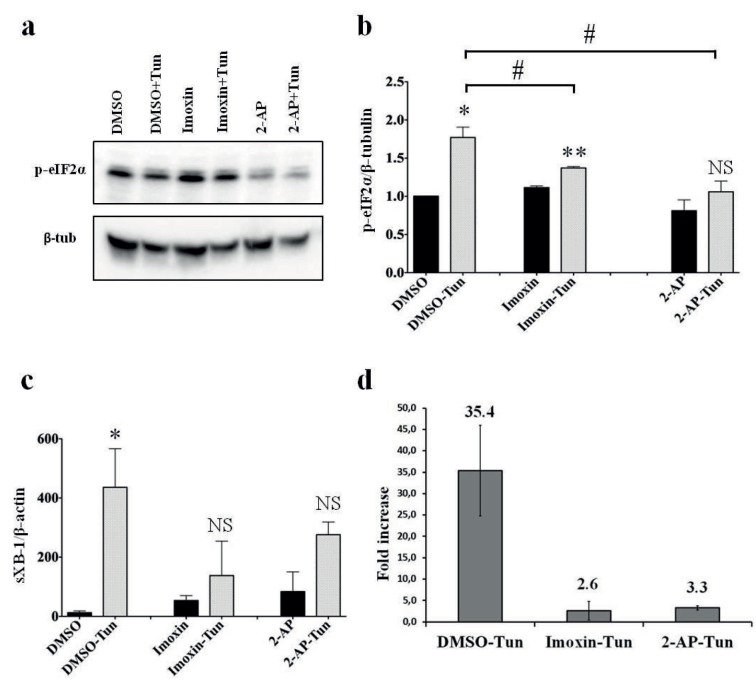
Suppression of ER stress by PKR inhibitors in pancreatic beta cells. a) Western blot analysis of elF2α phosphorylation (top picture) and β-tubulin (bottom picture) in PKR inhibitor (imoxin and 2-AP) pretreated Beta-TC-6 cells with and without tunicamycin (Tun) induction. b) Quantification of elF2α phosphorylation (normalized by β-tubulin expression) demonstrated by bar graph (*P < 0.05, **P < 0.005, NS: not significant). Comparison among tunicamycin-induced groups is indicated by horizontal lines (#P < 0.05). c) Expression of spliced XBP-1 mRNA in cell lysates (*P < 0.05, NS: not significant) normalized by actin mRNA expression. d) Fold increase in spliced XBP-1 mRNA expression in tunicamycin-induced Beta-TC-6 cells compared to their respective controls.

Tunicamycin increased phosphorylation of the serine residue of elF2α (Ser-51) compared to the controls, as expected, which is an indicator of ER stress (Figure 1a). In order to compare the severity of ER stress induction among different groups, the intensity of the protein bands of elF2α Ser-51 phosphorylation was quantified from independent experiments, normalized by the β-tubulin expression of the corresponding lanes (Figure 1b). Tunicamycin triggered significant increase in elF2α phosphorylation in imoxin and control (DMSO) pretreated cells compared to their respective controls; however, this increase was not statistically significant in 2-AP pretreated cells. When tunicamycin-treated Beta-TC-6 cells were compared to each other, a significant decrease in elF2α phosphorylation was observed in imoxin and 2-AP pretreated cells relative to DMSO pretreated cells (Figure 1b, horizontal lines), suggesting a mild suppression of ER stress by PKR inhibitor pretreatment. In order to further determine the ER stress status in the experimental groups by a second ER stress indicator, cytoplasmic splicing of XBP-1 mRNA was determined and quantified by real-time quantitative PCR using total cDNA from the cells as templates (Figure 1c). Likewise, tunicamycin induced a marked and significant increase in the XBP-1 mRNA splicing (normalized to actin mRNA) in DMSO pretreated cells. Although tunicamycin caused a similar increase in spliced XBP-1 mRNA in imoxin and 2-AP pretreated cells, these increases failed to reach statistical significance. As an indication of the extent of ER stress induction, fold increases in XBP-1 mRNA splicing among different tunicamycin-treated cells were determined (Figure 1d). Here we observed that, although tunicamycin induction increased the spliced XBP-1 mRNA 35.4-fold in DMSO pretreated cells (compared to uninduced controls), this increase was only 2.6- and 3.3-fold in imoxin and 2-AP pretreated cells, respectively. Overall, we observed a mild but significant suppression of ER stress in PKR inhibitor pretreated Beta-TC-6 cells upon tunicamycin induction.

### 3.2. The effect of PKR inhibition on beta cell insulin biosynthesis activity upon tunicamycin-induced ER stress

**Figure 2:**
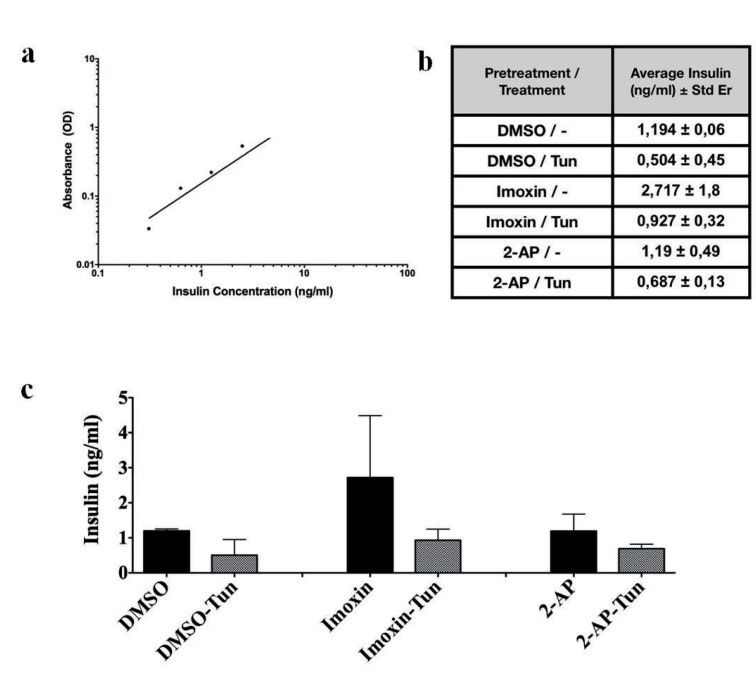
The effect of PKR inhibition on intracellular insulin content. a) Standard curve of known insulin concentration vs. absorption indication the efficiency of the assay. b) Intracellular insulin concentrations (ng per mL cell lysate) are shown (pretreatment/tunicamycin concentration 8 μg/mL). c) Bar graph of intracellular insulin concentrations (ng per mL cell lysate) is shown (pretreatment/tunicamycin concentration).

In order to determine whether suppression of ER stress by PKR inhibitors upon tunicamycin induction has any effect on beta cell insulin biosynthesis capacity, we pretreated Beta-TC-6 cells 24 h with imoxin, 2-AP, or DMSO, inducd ER stress by 8 μg/mL tunicamycin for 24 h, and finally prepared cell lysates, which were used to determine intracellular insulin content by insulin ELISA assay (Figure 2). Assay responsiveness and efficiency were determined by using a series of dilutions of recombinant insulin as described by the protocol (Figure 2a). The measured insulin concentrations in the lysates are indicated as ng/mL cell lysate (Figure 2b). Moderate decreases in the intracellular insulin were observed upon 8 μg/mL tunicamycin induction regardless of the pretreatment, although the decreases were not statistically significant (Figure 2c). Imoxin pretreatment slightly improved in control cells (uninduced cells); however, the effect was absent in 2-AP pretreatment (statistically not significant).

### 3.3. Induction of ER stress in pancreatic beta cells by free fatty acid toxicity in high-glucose environment

**Figure 3:**
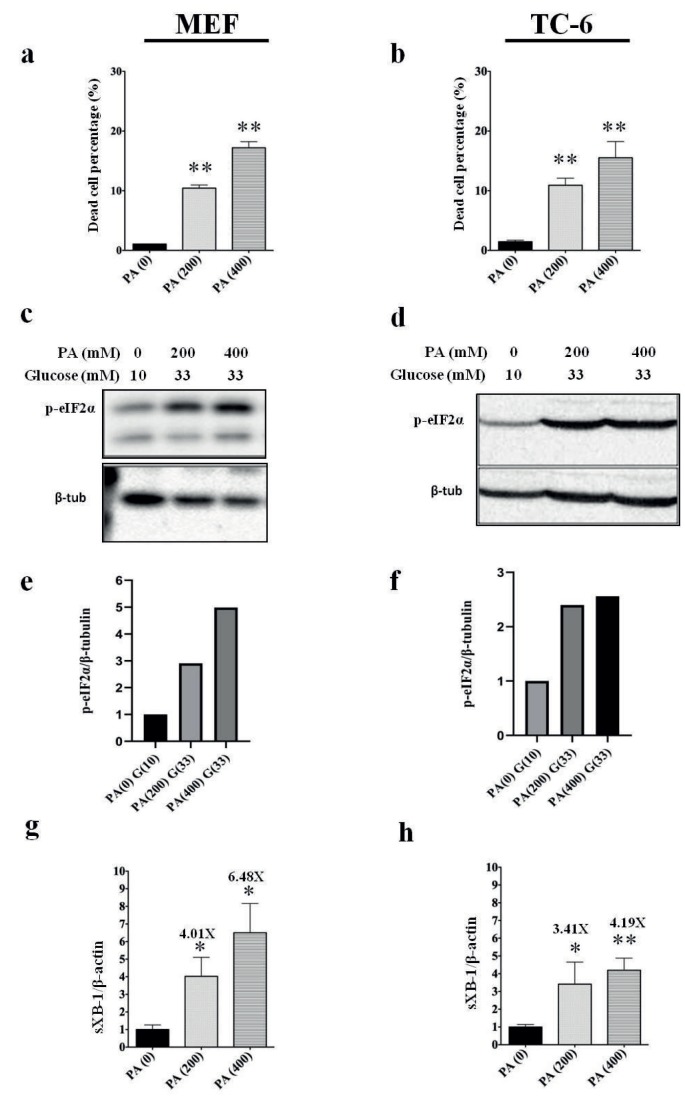
Induction of ER stress by PA in high-glucose environment. a) Percentage of dead cells in mouse embryonic fibroblast line (MEF) after PA treatment. (**P < 0.005). b) Percentage of dead cells in Beta-TC-6 cell line after PA treatment. (**P < 0.005). c) Western blot analysis of elF2α phosphorylation (top picture) and β-tubulin (bottom picture) in MEF cells by PA. d) Western blot analysis of elF2α phosphorylation (top picture) and β-tubulin (bottom picture) in Beta-TC-6 cells by PA. e, f) Quantification of elF2α phosphorylation (normalized by β-tubulin expression) demonstrated by bar graph. g) Expression spliced XBP-1 mRNA in MEF cell lysates after PA treatment (*P < 0.05) normalized by actin mRNA expression. h) Expression of spliced XBP-1 mRNA in Beta-TC-6 cell lysates after PA treatment (*P < 0.05, **P < 0.005) normalized by actin mRNA expression.

To determine the extent of cell death caused by PA, cells were suspended after the experiment and analyzed by cell counter. As expected, PA induced significant cell death in both cell lines tested (Figures 3a and 3b). In order to investigate the induction of ER stress in the cells, we determined Ser-51 phosphorylation of elF2α protein by western blot (Figures 3c and 3d) and spliced XBP-1 mRNA expression by real-time quantitative PCR (Figures 3e and 3f), using whole cell lysate proteins and cDNAs, respectively. In both cell lines tested, PA induced ER stress as evidenced by increased elF2α phosphorylation and increased spliced XBP-1 mRNA expression in a dose-dependent manner. Marked increases in elF2α phosphorylation suggest activation of elF2α kinases PERK and PKR by PA treatment.

### 3.4. Impairment of glucose-stimulated insulin secretion by glucolipotoxicity is subdued by repressing PKR activity

**Figure 4:**
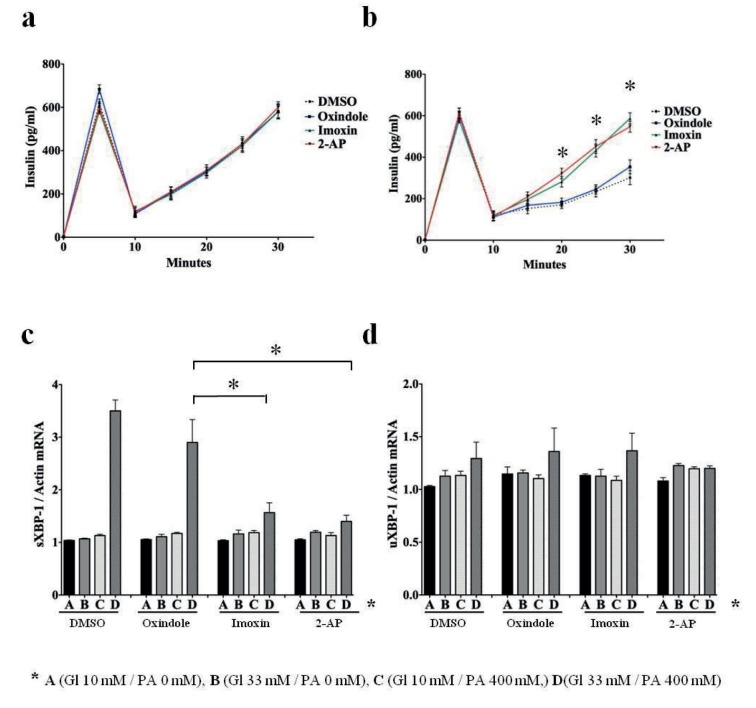
PA-mediated impairment of insulin secretion is subdued by PKR inhibitors. a) Glucose-stimulated insulin secretion assay of PA untreated Beta-TC-6 cells (GSIS) pretreated by DMSO, oxindole, imoxin, and 2-AP. Medium replenished 5 min after switching cells from no-glucose medium to high-glucose medium (33 mM glucose). b) GSIS assay of PA-treated (400 μM PA) Beta-TC-6 cells pretreated by DMSO, oxindole, imoxin, and 2-AP. Medium replenished 5 min after switching cells from no-glucose medium to high-glucose medium (33 mM glucose). Insulin concentrations of imoxin and 2-AP pretreated cells compared to oxindole pretreated cells are significantly different at 20, 25, and 30 min (*P < 0.05). c) Expression of spliced XBP-1 mRNA (sXBP-1) in cell lysates from corresponding cells (*P < 0.05) normalized by actin mRNA expression. d) Expression of unspliced XBP-1 mRNA (uXBP-1) in cell lysates from corresponding cells normalized by actin mRNA expression. PA and glucose concentrations of the treatment steps (a, b, c and d) are shown below.

In order to determine the effect of PKR inhibitors imoxin and 2-PA on PA-derived insulin secretion impairment in beta cells, we conducted a glucose-stimulated insulin secretion assay (GSIS) on the Beta-TC-6 cells after PKR inhibitor pretreatment for 24 h followed by PA treatment. To be able to determine the newly secreted insulin in the growth media, samples from the media were collected 0 and 5 min after the cells were switched to high-glucose medium (33 mM glucose), and the media were replenished with fresh high-glucose media to collect samples at 5-min intervals up to 30 min after the start of the assay (Figures 4a and 4b). Insulin concentrations in the media, which were below the detection limit of other assays, were determined by mouse ultrasensitive ELISA assay (ALPCO Cat#: 08-INSMSU-E01). Beta-TC-6 cells were pretreated for 24 h by PKR inhibitors imoxin, 2-AP, and DMSO before PA treatment. Imoxin and 2-AP are both imidazole-oxindole-derived molecules. Oxindole, which does not have any PKR inhibitory activity, was included as another negative control in the experiment (Ingrand et al., 2007). Beta-TC-6 cells that were not treated by PA did not display any impairment or betterment in insulin secretion efficiency with any pretreatment regime (Figure 4a). Cells that were treated by 400 μM PA in high-glucose medium (33 mM), on the other hand, displayed various levels of insulin secretion dependent on the pretreatment protocol (Figure 4b). Beta-TC-6 cells that were treated by DMSO and oxindole secreted significantly lower levels of insulin in the media at 20, 25, and 30 min of the experiment compared to the cells that were pretreated by PKR inhibitors imoxin and oxindole (P < 0.05). When the curves of different pretreatment protocols were compared by 2-way ANOVA analysis, the insulin secretion curves of imoxin and 2-AP appeared to be significantly different from both the DMSO and oxindole pretreatment curves, indicating that PKR inhibitors suppress insulin secretion impairment caused by PA treatment of Beta-TC-6 cells (Figure 4b). Finally, in order to determine the effect of PKR inhibitor pretreatments on the ER stress levels in Beta-TC-6 cells, we determined the expression of spliced and unspliced XBP-1 mRNA in the cell lysates after the GSIS assay (Figures 4c and 4d). Significant increase in spliced XBP-1 mRNA was detected in DMSO and oxindole pretreated cells by 400 μM PA in high-glucose medium (33 mM) compared to the PKR inhibitor pretreated cells (P < 0.05). Expression of unspliced XBP-1 mRNA did not appear to be increased by PA and was not affected by any pretreatment regime (Figure 4d). In summary, we demonstrated that PKR inhibitor pretreatment appears to suppress PA-mediated impairment of insulin secretion from pancreatic beta cells by suppressing ER stress.

## 4. Discussion

The discovery of new therapeutics targeting different molecules has become very crucial in recent years to overcome the increasing prevalence of metabolic diseases such as diabetes and obesity. Metabolic inflammation, also known as metaflammation, is the leading complication of these diseases. Previously, it has been shown that inflammatory pathways are triggered and that insulin signaling is altered by PKR (Nakamura et al., 2010; Carvalho-Filho et al., 2012), so the suppression of the PKR pathway may be a novel target of treatment. Here, we examined the effects of PKR inhibitors on ER stress markers and insulin secretion in ER-stressed cells. Our findings suggest that PKR inhibitors are effective in ameliorating ER stress and improving insulin secretion. 

Many lipotoxic and some ER stress inducer-mediated models have demonstrated that the association of ER stress with beta cell apoptosis is via the CHOP protein, one of the downstream targets of IRE1 (Åkerfeldt et al., 2008; Cunha et al., 2008; Song et al., 2008). However, the main reason for ER stress-induced cell death in beta cells is the accumulation of lipids in peripheral tissues (Cunha et al., 2008). Besides, as we found in our study, it has been shown that the intermediates of fatty acids degraded by β-oxidation become much more harmful with high glucose (Poitout et al., 2010). It will be interesting to see whether the blocking of PKR activation by small molecules also protects beta cells from ER stress-mediated apoptosis.

UPR provides phosphorylation of eIF2α to inhibit general protein translation in order to reduce the damage caused by protein overload in ER (Youssef et al., 2015). This process links the PKR and UPR pathways. ER stress marker expressions have also been shown to increase significantly in diabetic mice and T2D patients (Laybutt et al., 2007; Marchetti et al., 2007). Additionally, increased blood sugar and saturated/unsaturated fatty acids, especially palmitate, have been found to impair insulin signaling, cause PERK-mediated phosphorylation of eIF2α, induce IRE1, and promote UPR in beta cells by splicing XBP-1 protein (Cnop et al., 2007; Gwiazda et al., 2009). It was known to us from the published work that PA induces ER stress in a cultured mouse embryonic fibroblast cell line. Therefore, we used mouse embryonic fibroblasts as a positive control for a PA-induced ER stress model (Guo et al., 2007). In our study, we also found that eIF2α phosphorylation and XBP splicing did not occur with high glucose but rather with PA and glucose coexposed cells. A previous study indicated that PKR inhibitor imoxin suppresses PKR-mediated eIF2α phosphorylation and provides a significant increase in insulin receptor substrate (IRS) expression in vitro. In the same study, it was found that PKR inhibitors (imoxin and 2-aminopurine) lower blood glucose and improve the insulin sensitivity of genetically obese mice (Nakamura et al., 2014). In our study, imoxin or 2-AP alone did cause a significant increase in eIF2α phosphorylation and Xbp-1 mRNA splicing in tunicamycin-untreated cells. The reason for these effects was not determined. However, according to our observations, PKR inhibition in tunicamycin-untreated cells did not cause any detectable cell death or visible morphological changes in a way that would change the conclusions of our findings. In this study we demonstrated that PKR inhibition may have additional benefits against metabolic malformations in diabetic patients through improving pancreatic insulin secretion capacity. Our findings suggest that PKR inhibitors may be used in the treatment of type 2 diabetes mellitus in later stages in order to ameliorate pancreatic beta cell degeneration.

## Acknowledgments

This work was supported by the Scientific and Technological Research Council of Turkey (TÜBİTAK Grant 115C106) and Aydın Adnan Menderes University Scientific Research Projects Grant TPF-17059.
